# Neoprene Orthopaedic Supports: An Underrecognised Cause of Allergic Contact Dermatitis

**DOI:** 10.1155/2015/496790

**Published:** 2015-07-07

**Authors:** S. Hawkey, S. Ghaffar

**Affiliations:** Department of Dermatology, Ninewells Hospital & Medical School, University of Dundee, Dundee DD1 9SY, UK

## Abstract

Thioureas, often contained within neoprene to provide water resistance, are an important cause of allergic contact dermatitis (ACD) in those who use neoprene products. We wish to present three cases of thiourea-induced ACD from three different orthopaedic supports containing neoprene. The first case was a 67-year-old woman who developed an itchy rash on her heel three weeks after using a neoprene insole for plantar fasciitis. The second case was a 47-year-old man who developed an itchy rash on his wrist after wearing neoprene wrist splints for psoriatic arthropathy. The third case was a 77-year-old woman who experienced a severe erythematous rash with blistering from a neoprene elbow brace she received following a humeral fracture. All patients were patch tested to the British Society of Cutaneous Allergy Standard and rubber series and a cut piece from all the relevant supports. At 96 hours, all patients had a + reaction to mixed dialkylthiourea, diethylthiourea, and the supports' material. No other positive patch test reactions were identified. As neoprene is fast becoming one of the most popular materials used for orthopaedic supports, awareness of this reaction and close liaison between dermatologists and orthopaedic surgeons are therefore essential to allow for early recognition of this complication.

## 1. Introduction

Neoprene is a popular material widely used in a variety of products including wet suits and computer mouse pads [1]. Due to its soft texture, controlled stretch, and good cosmesis, neoprene is also now becoming one of the most popular materials used for a range of orthopaedic supports including braces, insoles, and splints [2–5]. However, thiourea compounds contained within neoprene can trigger allergic contact dermatitis (ACD). We wish to present three cases of ACD triggered by thioureas contained within three different types of neoprene orthopaedic support.

## 2. Patient Cases

**Figure 1:**
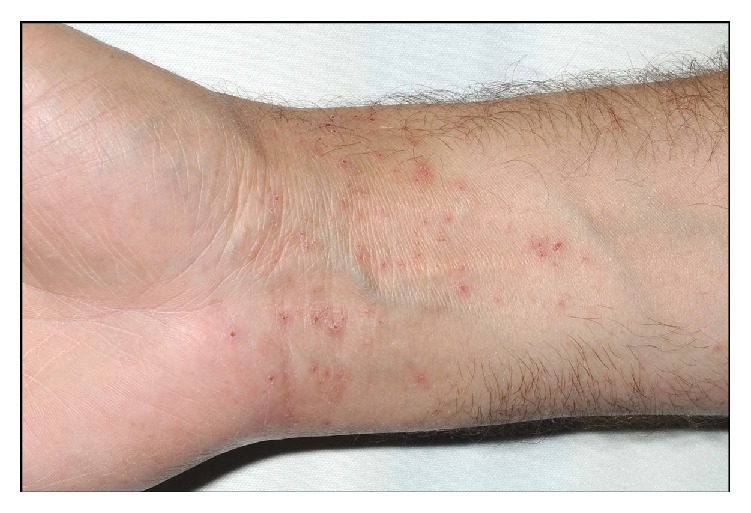
Itchy reaction to a wrist splint used for psoriatic arthropathy.

**Figure 2:**
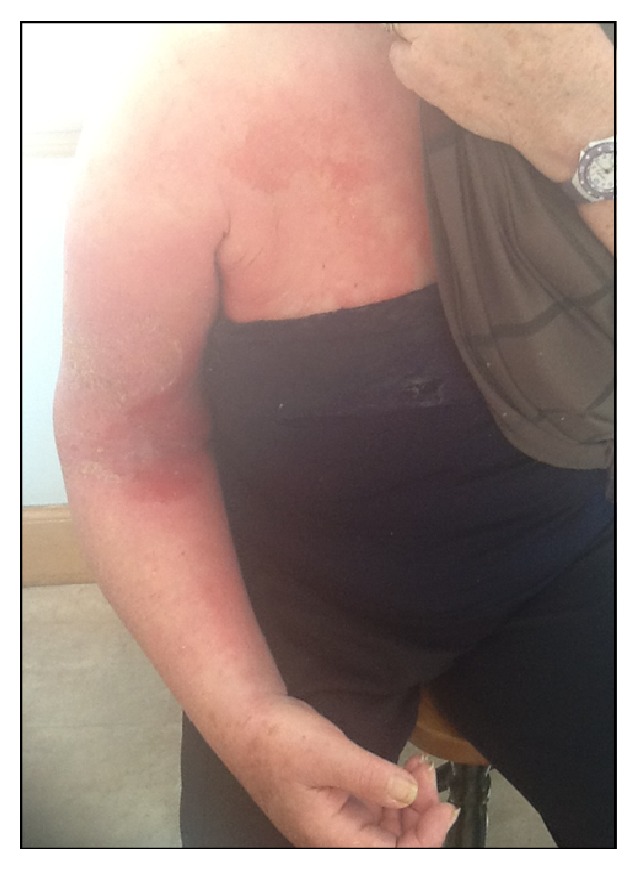
Erythematous blistering rash over the skin in contact with the brace material.

**Figure 3:**
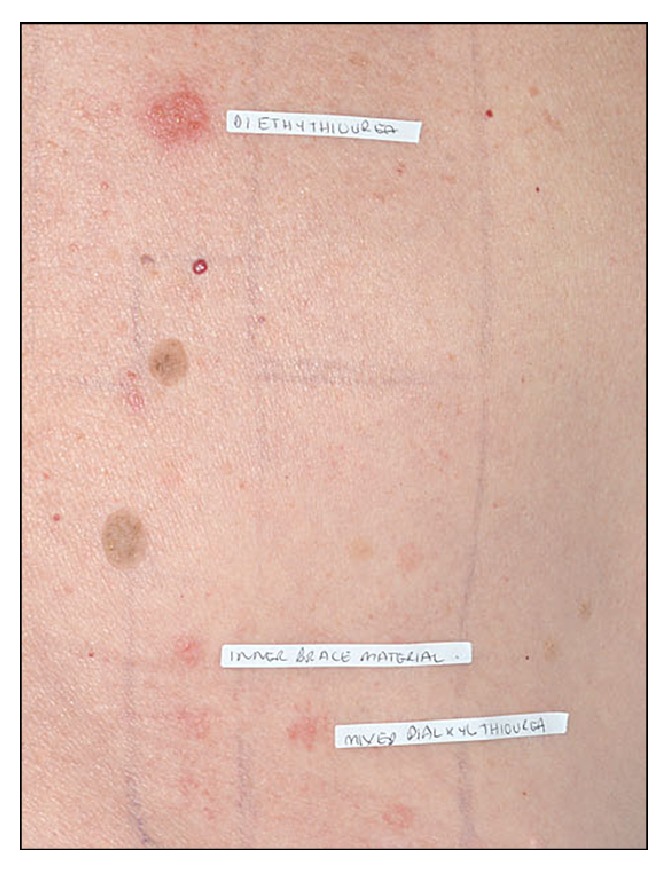
Positive patch test reaction to mixed dialkylthiourea, diethylthiourea, and the supports' material.

The first case was a 67-year-old lady who presented with an itchy dermatitis on her heel three weeks after she used a neoprene insole as treatment for her plantar fasciitis. The second case was a 47-year-old man who developed an itchy rash on his wrist after wearing three different types of neoprene wrist splints for his psoriatic arthropathy (Figure 1). The rash would typically appear 24–48 hours after he started wearing each splint. The third case was a 77-year-old lady who fractured her humerus and was given a neoprene elbow brace. A month after she started using it, she developed a severe, itchy erythematous rash with blistering over the skin in contact with the brace material (Figure 2). All three patients were patch tested to the British Society of Cutaneous Allergy (BSCA) Standard and rubber series along with a cut piece from all the relevant supports. At 96 hours, all three patients had a + reaction to mixed dialkylthiourea, diethylthiourea, and the supports' material (Figure 3). The BSCA Standard and rubber series encompasses a range of materials including common preservatives, dyes, excipients of topical medicaments, and rubber accelerators. In all three patients, no other positive patch test reactions were identified.

## 3. Discussion

Neoprene, developed in 1931, is one of the earliest synthetic rubbers. Thioureas are a group of chemicals used to accelerate the curing process that creates neoprene rubber and helps provide its water resistant properties [6]. The first case of thiourea allergy was only reported in the late 1960s despite its increasing use in industry since the beginning of the last century [7, 8]. Since then, an increasing number of thioureas containing neoprene products have been reported to cause allergic contact dermatitis [6, 9]. As cited by previous authors, there are several thiourea compounds that can be used in neoprene so an individual has the potential to react to one or several of these compounds [2]. Furthermore, as labelling of individual composition of neoprene products is not compulsory, it will be difficult for allergic patients to know which to avoid.

The general prevalence of positive patch test reactions to mixed dialkylthiourea has been reported as high as 2.4% [10], and the rise in neoprene use within orthopaedics means it is likely that this will become an increasing problem within the specialty. As was evident in our cases, ACD to neoprene can cause troublesome symptoms including severe itching, eczematous eruptions, and in some cases blistering, meaning it is an important condition to recognise and manage correctly as it may be misdiagnosed and incorrectly treated. Before providing patients with any neoprene-containing support, we encourage clinicians to ask patients about previous reactions to neoprene products. In patients who describe a history of this, alternatives should be considered and patients should be referred for patch testing to confirm the allergy. Furthermore, it is important to educate patients on the potential risk of ACD associated with neoprene so that they can inform clinicians early should they notice any signs of reaction.

Although each patient described an adverse skin reaction to their support and had positive patch tests to the supports material and to thioureas, we were not able to prove directly that thioureas were contained within each of the orthopaedic supports. We had written to each of the companies who supplied the supports but two of these did not reply, and one company stated they were not able to provide information regarding the material used. Nevertheless, the combination of these three reactions described above makes a causal connection highly likely in these patients.

## 4. Conclusion

Our cases highlight an important complication of neoprene support use in a variety of orthopaedic treatment modalities. Awareness of this potential reaction and close liaison between dermatologists, orthopaedic surgeons, rheumatologists, occupational therapists, and physiotherapists are therefore essential so as to allow for early recognition and appropriate investigation to be undertaken.
